# Diets shape thermal responses in Chinese giant salamanders by altering liver metabolism

**DOI:** 10.3389/fmicb.2025.1546912

**Published:** 2025-03-18

**Authors:** Runliang Zhai, Chunlin Zhao, Liming Chang, Jiongyu Liu, Tian Zhao, Jianping Jiang, Wei Zhu

**Affiliations:** ^1^Chengdu Institute of Biology, Chinese Academy of Sciences, Chengdu, China; ^2^University of Chinese Academy of Sciences, Beijing, China; ^3^School of Biological and Chemical Engineering (School of Agriculture), Panzhihua University, Panzhihua, China; ^4^College of Fisheries, Southwest University, Chongqing, China

**Keywords:** conservation, energy metabolism, gut microbiota, metabolomics, metagenomics, warming

## Abstract

Diet can influence the thermal performance of ectotherms, providing potential strategies for biological conservation in the context of global warming. The endangered *Andrias davidianus* is susceptible to heat stress due to energy deficiency in the liver when fed a worm-based diet rich in carbohydrates. A fish-based diet, rich in protein and lipids, improves their thermal performance, but the underlying physiological mechanisms remain unclear. In this study, we used metabolomics and metagenomics to examine the combined effects of temperature (15, 20, and 25°C) and diet (fish-based and worm-based) on liver metabolism and gut microbiota. Our results show that both temperature and diet shape liver metabolism, with several vital metabolic pathways (e.g., TCA cycle and sulfate metabolism) regulated by their combined effects. Notably, diet-dependent thermal responses in energy metabolism were observed, with fish-fed salamanders exhibiting a marked upregulation of the TCA cycle intermediates under heat stress, a response absent in worm-fed individuals. Given the role of TCA cycle in heat susceptibility of *A. davidianus*, these findings suggest that the TCA cycle likely mediates the interactive effects of temperature and diet on thermal performance. We then examined whether the gut microbiota is also a target of interactive effects or a mediator of the diet’s influence on liver metabolism. While both temperature and diet shape microbiota composition, functional shifts occur only in response to temperature, indicating that the microbiota is not a major link between diet and liver metabolism. However, several bacterial groups (e.g., *Thiosulfatimonas* and *Alcanivorax*), jointly regulated by temperature and diet, correlate with liver metabolites, suggesting alternative, function-independent pathways through which dietary-related microbial changes may influence liver metabolism and even thermal tolerance. Overall, this study provides molecular insights into the dietary modulation of thermal performance in *A. davidianus* and highlight the potential of dietary microbial management strategies for amphibian conservation.

## 1 Introduction

Warming poses a significant threat to animal health and productivity, leading to functional impairments, metabolic exhaustion, oxidative damage, immune suppression, and accelerated aging (Wang J.-H. et al., 2024). Ectotherms are particularly vulnerable to changes in environmental temperature due to their narrow thermal tolerance ranges and temperature-dependent metabolic rates (Deutsch et al., 2008; Jørgensen et al., 2022). Among vertebrates, amphibians are especially sensitive to environmental changes because of their strong reliance on specific conditions to complete their life cycles (Lötters et al., 2023; Mi et al., 2023; Wu et al., 2024), placing 40.7% of species globally at risk (Luedtke et al., 2023). Warming is widely recognized as a major driver of the ongoing decline in wild amphibian populations (Alan Pounds et al., 2006; Alford et al., 2007; D’Amen and Bombi, 2009). Understanding the factors that influence amphibian thermal tolerance and adaptability is critical for developing conservation strategies and prioritizing efforts to protect the most vulnerable species (Hofmann and Todgham, 2010; Gonzalez-Rivas et al., 2020).

Diet has been shown to significantly influence the thermal performance of ectotherms, including their thermal limits and preferences, in taxa such as insects (Hardison et al., 2021; Wang Y. et al., 2024), amphibians (Zhao et al., 2022), reptiles (Geiser et al., 1992), and particularly fish (Abdel-Ghany et al., 2019; Gomez Isaza et al., 2019; Ciji et al., 2021; Hardison et al., 2023). For instance, dietary supplementation with L-tryptophan has been demonstrated to enhance thermal tolerance and reduce oxygen consumption in *Cirrhinus mrigala* fingerlings (Tejpal et al., 2014). The macronutrient and micronutrient composition of diets influences ecologically significant traits such as metabolism, growth, digestion, and cardiac function, which are involved in an organism’s response to temperature changes and subsequently shape its thermal performance (Kumar et al., 2016; Richard et al., 2016; Hardison and Eliason, 2024). These findings suggest that dietary composition may play a pivotal role in modulating the thermal adaptability of animals, with potential applications for conservation strategies targeting key species under climate change scenarios. However, research on the relationship between diet and thermal performance in amphibians remains far less extensive than in fish.

Exploring the molecular basis of the interactions between temperature and diet could greatly enhance the application of this knowledge to species conservation in the context of global climate change. Two key physiological pathways may underlie the interaction between diet and temperature in shaping the thermal physiology of animals (Hardison and Eliason, 2024). First, dietary components can directly influence metabolic patterns, either by serving as metabolic substrates or by acting as regulators. For example, nutrient-rich diets provide the energy required for cellular repair, stress responses, and metabolic demands under thermal stress (Bujan and Kaspari, 2017), while dietary fatty acid profiles are critical for membrane adaptation to temperature changes (Alhazzaa et al., 2013). Second, the gut microbiome may mediates the effects of diet shift on thermal performance. Both factors strongly influence the physiology, community composition, and metabolic profiles of host-associated microbiota (Bestion et al., 2017; Soriano et al., 2018; Trevelline et al., 2019; Ayayee et al., 2020; Zhu W. et al., 2021; Zhu et al., 2024), which in turn modulate the host’s thermal physiology (e.g., thermal tolerance) (Gupta et al., 2010; Moeller Andrew et al., 2020; Fontaine et al., 2022). For example, translocating gut microbiomes between frog species enhances host heat tolerance, likely due to increased short-chain fatty acids and reduced pathogens (Dallas et al., 2023). These pathways offer distinct molecular mechanisms, providing a framework for understanding how diet and temperature interact to influence thermal physiology. For a given species, determining the relative importance of these pathways is essential for developing effective conservation strategies (Trevelline et al., 2019).

The endangered Chinese giant salamander (*Andrias davidianus*), one of the largest extant amphibians, is a flagship species for amphibian conservation (Yan et al., 2018; Shu et al., 2021). Temperature is a critical environmental factor influencing the physiological performance of *A. davidianus* (Zhang et al., 2014; Hu et al., 2019; Zhu et al., 2021b; Zhao et al., 2022). Although this species demonstrates notable cold tolerance (Zhu et al., 2022a), it is highly susceptible to temperatures exceeding 20°C, which result in decreased feeding activity, accelerated development, suppressed growth, and increased mortality (Chen et al., 1999; Wang, 2004; Mu et al., 2011; Hu et al., 2019). For instance, our previous study found that *A. davidianus* exhibited poor growth performance at 25°C when fed a worm-based diet (rich in carbohydrates but low in proteins and lipids) (Zhu et al., 2022b). This was accompanied by decreased gut microbial alpha diversity and compositional changes (Zhu et al., 2021b). Interestingly, the growth performance of heat-stressed larvae improved when they were switched to a fish-based diet rich in proteins and lipids. Moreover, individuals fed the fish diet exhibited higher upper thermal tolerance (the temperature at which larvae escape or lose balance) compared to those on the worm diet (Zhao et al., 2022). These findings underscore the critical role of diet composition in shaping the thermal performance of *A. davidianus*. Further analyses revealed that the liver plays a pivotal role in poor thermal performance under heat stress, characterized by morphological enlargement, energy deficiency, reduced protein levels, and decreased TCA cycle intermediates due to impaired glycogen metabolism (Zhu et al., 2022b). These molecular and metabolic insights provide a robust foundation for investigating how diet influences the thermal adaptability of *A. davidianus*.

**Figure 1:**
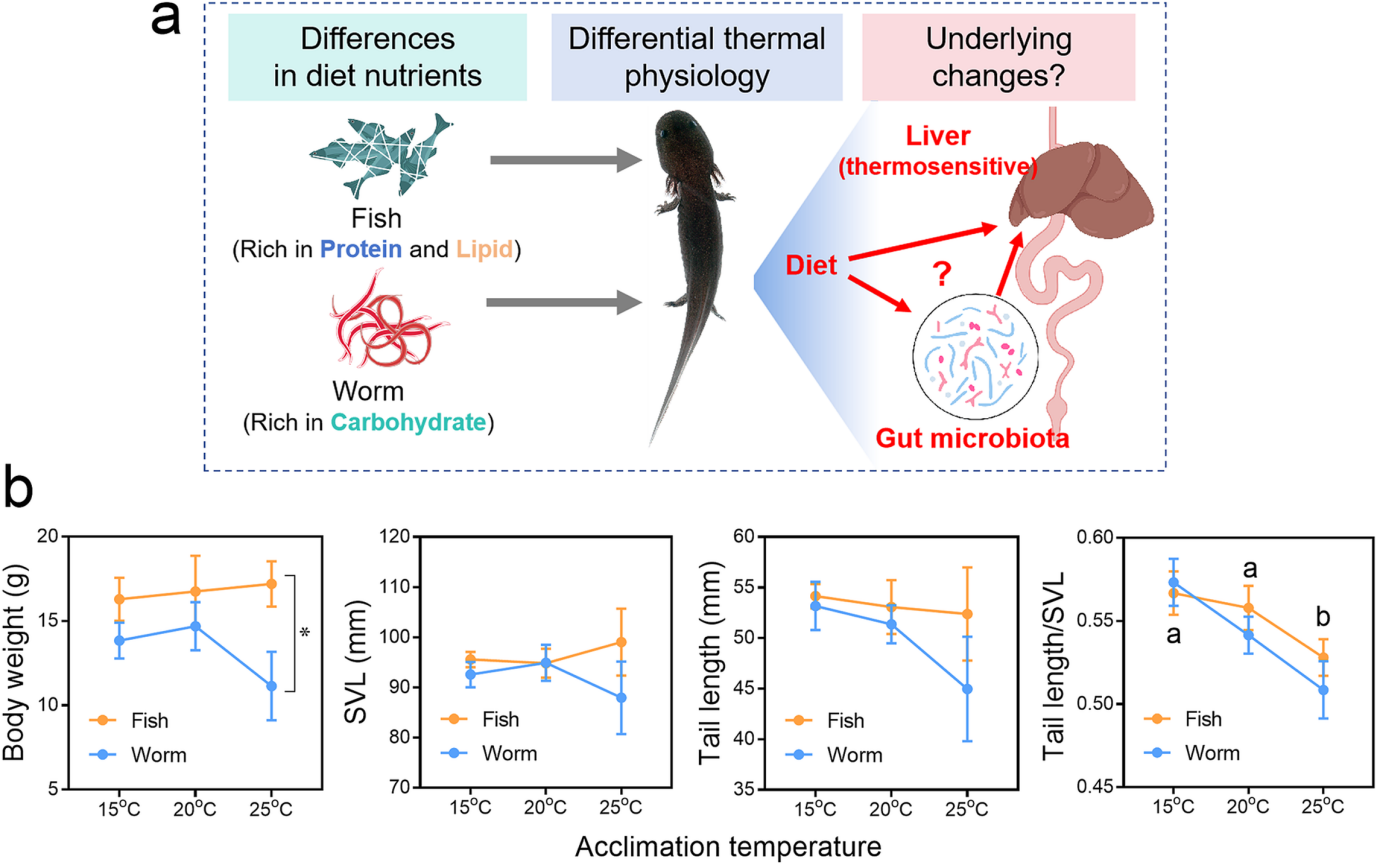
Effects of diet on the thermal physiology of *Andrias davidianus*. **(a)** Schematic diagram depicting the scientific questions addressed in this study. **(b)** Body traits of *A. davidianus* larvae acclimated to different temperatures for 90 days. Asterisks indicate significant differences between diet groups, while different letters indicate significant differences between temperature groups (*p* < 0.05, two-way ANOVA with S-N-K *post hoc* tests).

In this study, we employed metabolomics and metagenomics to investigate the effects of temperature (15, 20, and 25°C) and diet (fish-based and worm-based) on the liver metabolome and gut microbiome functions of *A. davidianus*. Our objective was to elucidate the mechanisms by which diet influences the thermal tolerance of this species. We hypothesized that (1) temperature and diet interact or act additively to affect liver metabolism and gut microbial functionality in *A. davidianus*, and (2) the liver metabolic pathways or gut microbial functions targeted by both temperature and diet overlap with key metabolic pathways (e.g., energy metabolism) associated with thermal sensitivity in *A. davidianus* (Figure 1a). This research aims to deepen our mechanistic understanding of the dietary influence on amphibian thermal physiology, providing valuable insights for thermal physiology theory and its applications in conservation biology.

## 2 Materials and methods

### 2.1 Animals and thermal acclimation

Larvae of the giant salamander (Shanxi clade, at first year of age, body weight = 13.3 ± 0.34 g; mean ± se) were collected from a semi-natural aquaculture farm (102^o^10’05″ E, 29 ^o^52’36” N) located in Hongya County, Sichuan Province, China, where the water temperature was maintained between 13 and 17°C (Zhu et al., 2022a). Prior to treatment, the larvae were maintained at an air temperature of 17 ± 1.2°C (12 L, 12D) and fed red worms (*Chironomus* sp. larvae) for 2 weeks to acclimate them to artificial conditions. The larvae were then randomly assigned to six groups: 15°C + fish diet (chopped), 20°C + fish diet, 25°C + fish diet, 15°C + worm diet, 20°C + worm diet, and 25°C + worm diet (air temperature). The choice of temperature conditions is informed by our previous research on the thermal adaptability and heat sensitivity of *A. davidianus*, ensuring that this study remains comparable to our earlier work (Zhu et al., 2021b; Zhao et al., 2022; Zhu et al., 2022a; Zhu et al., 2022b). Additionally, 15°C is empirically recognized as the optimal temperature for *A. davidianus*, while 20°C and 25°C are widely regarded as heat stress temperatures for the species (for more detailed information, please refer to our earlier publications). The nutrient composition of the worm diet consisted of 1.21% lipids, 5.11% protein, and 1.65% carbohydrates, while the fish diet provided 8.66% lipids, 17.7% protein, and 0.39% carbohydrates. The total protein content was determined using the Kjeldahl method, as outlined in China national standard GB 5009.5–2016; the total carbohydrate content was measured using the phenol method according to GB/T 9695.31–2008; and the total lipid content was determined via the acid hydrolysis method specified in GB 5009.6–2016. The results are presented as the percentage of each nutritional component’s weight relative to the total wet weight. The selection of these two diets is based on our previous research examining the effect of diet on the thermal performance of *A. davidianus* (Zhao et al., 2022). These feeds are commonly used in farms and are also the standard diets for maintaining *A. davidianus* in our laboratory. These groups were housed in three climate chambers set to different temperatures. Each group contained three plastic containers (35 cm × 25 cm × 15 cm, with a water depth of 5 cm), representing three replicates, with five individuals per replicate. The treatment lasted 88 days, during which the water was changed, and the larvae were provided with an adequate amount of either the worm or fish diet every 2 days. The larvae were measured for the body weight, snout-vent length (SVL), and tail length at the end of the experiment. After euthanized using MS-222 in water, the larvae were dissected to collect the liver tissue and gut content. All animal protocols in this study were reviewed and approved by the Animal Ethical and Welfare Committee of the Chengdu Institute of Biology, Chinese Academy of Sciences (permit number: CIBDWLL2023013), in compliance with the ARRIVE guidelines 2.0 (Percie du Sert et al., 2020) and Guide for the Care and Use of Laboratory Animals (8th edition) published by National Research Council (US) Committee for the Update of the Guide for the Care and Use of Laboratory Animals (2011).

### 2.2 Metagenomic analyses

Gut contents of the larvae were collected for metagenomic analysis, with six samples obtained from each group. Samples at 25°C were excluded from both gut metagenome and liver metabolome analyses due to high mortality at this temperature (regardless of diet). The surviving individuals at this temperature were likely in an abnormal physiological state. Total genomic DNA was extracted using the Zymo Research BIOMICS DNA Microprep Kit (Zymo Research Co., U.S.). Nucleic acid concentrations were measured on a Tecan F200 (Tecan, Switzerland) using the PicoGreen assay. DNA fragmentation was performed with a Covaris M220 (Covaris, United States), followed by library preparation using the NEBNext^®^ Ultra™ II DNA Library Prep Kit and NEBNext^®^ Multiplex Oligos for Illumina^®^ (Dual Index Primers Set 1) (New England Biolabs, United States). Library quality was evaluated with an Agilent 2,100 Bioanalyzer (Agilent Technologies, United States), and concentrations were quantified by qPCR. Sequencing was carried out on an Illumina NovaSeq 6,000 platform (Rhonin Biosciences, China). Raw data were deposited in the Genome Sequence Archive (GSA) under the accession number CRA021169 (Chen et al., 2021; CNCB-NGDC Members and Partners, 2021).

Raw sequences were processed with Trimmomatic to remove low-quality bases and sequencing adapters (parameters: LEADING: 5, TRAILING: 5, SLIDINGWINDOW: 4:15, and MINLEN: 100) (Bolger et al., 2014). Quality assessments were performed on the sequences before and after trimming using fastQC and MultiQC (Andrews, 2010; Ewels et al., 2016). Sequence assembly for each sample was conducted using Megahit (Li et al., 2016), and the assembled sequences were evaluated for quality using Quast (Gurevich et al., 2013). Gene prediction on the assembled contigs was performed using Prodigal (Hyatt et al., 2010). All genes across samples were pooled, and gene set dereplication was carried out using MMseq2 (with a coverage threshold of 80% and similarity threshold of 90%) (Mirdita et al., 2020). The Burrows-Wheeler Alignment Tool was used to map the clean reads back to the non-redundant gene set, enabling the evaluation of sequence counts for each non-redundant gene in each sample and subsequent gene quantification (Li, 2013). Functional annotation of the non-redundant gene set was performed using databases NR and KEGG (Kanehisa et al., 2011; Coordinators, 2017; Terrapon et al., 2017; Huerta-Cepas et al., 2018; Liu et al., 2018; Alcock et al., 2019; Urban et al., 2019). Community structure analysis was conducted using Kraken based on the RefSeq database (O'Leary et al., 2016; Wood et al., 2019). The alpha-diversity indices (e.g., Shannon index) were calculated using *diversity* function in R package vegan (Dixon, 2003).

### 2.3 Metabolome analyses

For each liver sample (*n* = 6 for each group), approximate 50 mg liver tissue was weighed before the extraction of metabolites and dried lyophilized were ground in a 2 mL Eppendorf tube containing a 5 mm tungsten bead for 1 min at 65 Hz in a Grinding Mill. Metabolites were extracted using 1 mL precooled mixtures of methanol, acetonitrile and water (v/v/v, 2:2:1) and then placed for 1 h ultrasonic shaking in ice baths. Subsequently, the mixture was placed at −20°C for 1 h and centrifuged at 14,000 *g* for 20 min at 4°C. The supernatants were recovered and concentrated to dryness in vacuum.

Metabolomic profiling was conducted by Bioprofile (Shanghai, China) using a UPLC-ESI-Q-Orbitrap-MS system, consisting of a Shimadzu Nexera X2 LC-30 AD UHPLC (Shimadzu, Japan) coupled with a Q-Exactive Plus mass spectrometer (Thermo Scientific, San Jose, United States). For liquid chromatography (LC) separation, an ACQUITY UPLC^®^ HSS T3 column (2.1 × 100 mm, 1.8 μm) (Waters, Milford, MA, United States) was used. The flow rate was 0.3 mL/min, and the mobile phase consisted of: (A) 0.1% formic acid (FA) in water and (B) 100% acetonitrile (ACN). The gradient profile was as follows: 0% buffer B for 2 min, increasing linearly to 48% buffer B over 4 min, then ramping up to 100% buffer B in another 4 min, maintaining 100% buffer B for 2 min, and decreasing to 0% buffer B in 0.1 min, followed by a 3-min re-equilibration period. The HESI source conditions were set as follows: Spray voltage: 3.8 kV (positive) and 3.2 kV (negative); Capillary temperature: 320°C; Sheath gas (nitrogen) flow: 30 arb (arbitrary units); Aux gas flow: 5 arb; Probe heater temperature: 350°C; S-Lens RF level: 50. The instrument was set to acquire over the m/z range of 70–1,050 Da for full MS scans. Full MS scans were performed at a resolution of 70,000 at m/z 200, and MS/MS scans were acquired at a resolution of 17,500 at m/z 200. The maximum injection time was set to 100 ms for MS and 50 ms for MS/MS. The isolation window for MS2 was set to 2 m/z, and the stepped normalized collision energy was set to 20, 30, and 40 for fragmentation. Quality control (QC) samples were prepared by pooling aliquots from all individual samples, ensuring they represented the overall sample set, and were used for data normalization. Blank samples (75% ACN in water) and QC samples were injected every six samples during acquisition.

The raw MS data were processed using MS-DIAL for peak alignment, retention time correction and peak area extraction. The metabolites were identified by accuracy mass (mass tolerance <10 ppm) and MS/MS data (mass tolerance <0.02 Da) which were matched with HMDB, massbank and other public databases and our self-built metabolite standard library. In the extracted-ion features, only the variables having more than 50% of the non-zero measurement values in at least one group were kept.

### 2.4 Statistical analyses

Basic statistical analyses were conducted using IBM SPSS v21.0 (IBM, Armonk, NY, United States) and R (R Core Team, 2021). Kolmogorov–Smirnov and Levene’s tests were employed to examine the distribution and homoscedasticity of the data. The influences of temperature and diet on the body traits and microbial alpha-diversity of *A. davidianus* larvae were examined using two-way ANOVA and S-N-K *post hoc* tests, with a significance threshold of *p* < 0.05. Bray-Curtis distance matrixes were calculated for microbial diversity and functional data, as well as tissue metabolic data (Dixon, 2003), representing the dissimilarity between samples. These distance matrices can be used for permutational multivariate analysis of variance (PERMANOVA) to assess whether significant differences exist between the two groups at the overall microbiome or metabolomic profile level. Principal coordinates analysis (PCoA) was used to visualize these dissimilarities. PERMANOVA was conducted to assess whether samples from different groups showed significant differences in their overall microbiome or metabolome, with a threshold of adjusted *p* < 0.05 (after Benjamini and Hochberg’s (BH) correction for multiple comparisons) (Dixon, 2003). Specifically, a multi-factor PERMANOVA with diet and temperature as factors (type II sum of square) was performed to examine potential significant interactions and to determine which factor account for a greater proportion of the variance (*R*^2^ value). This is followed by differential analyses to screen differential items between groups. If a significant interaction (*p* < 0.05) between diet and temperature is observed (PERMANOVA), pairwise comparisons between temperature groups within each diet condition are conducted using Student’s *t*-tests, and vice versa. If the interaction is not significant (*p* > 0.05), we use two-way ANOVA for subsequent differential analyses. The thresholds of differential analyses were set at both *p* < 0.01 and adjusted *p* < 0.05 (BH corrections). This strategy helps reduce the risk of false negatives that may arise from the large number of bacterial groups or metabolites during BH correction. Metabolite enrichment analyses were performed using MetaboAnalyst 6.0^1^. Mantel tests were conducted to examine the potential associations between metagenome and metabolome of the *A. davidianus* larvae at a whole level, with a threshold of *p* < 0.05. Spearman correlation and BH corrections were performed to screen the correlations in the relative abundance between individual metabolites and microbial taxa or functional pathway, with a threshold of adjusted *p* < 0.05 and absolute *R* > 0.75. Graphs were generated using GraphPad Prism 7 (San Diego, CA, United States), Cytoscape (Shannon et al., 2003), MedPeer (medpeer.cn), and ggplot2 (Wickham, 2016).

## 3 Results

Morphological traits of *A. davidianus* larvae were measured at the end of the experimental treatments (Figure 1b). No significant differences in body weight, snout-to-vent length (SVL), or tail length were detected among individuals exposed to different temperatures. However, larvae reared at 25°C exhibited a reduced tail-to-SVL ratio, suggesting diminished fat storage, consistent with previous findings (Zhu et al., 2022b). Regarding dietary effects, fish-fed individuals displayed significantly higher body weights than those fed a worm-based diet.

### 3.1 Effects of temperature and diet on the liver metabolome

**Figure 2:**
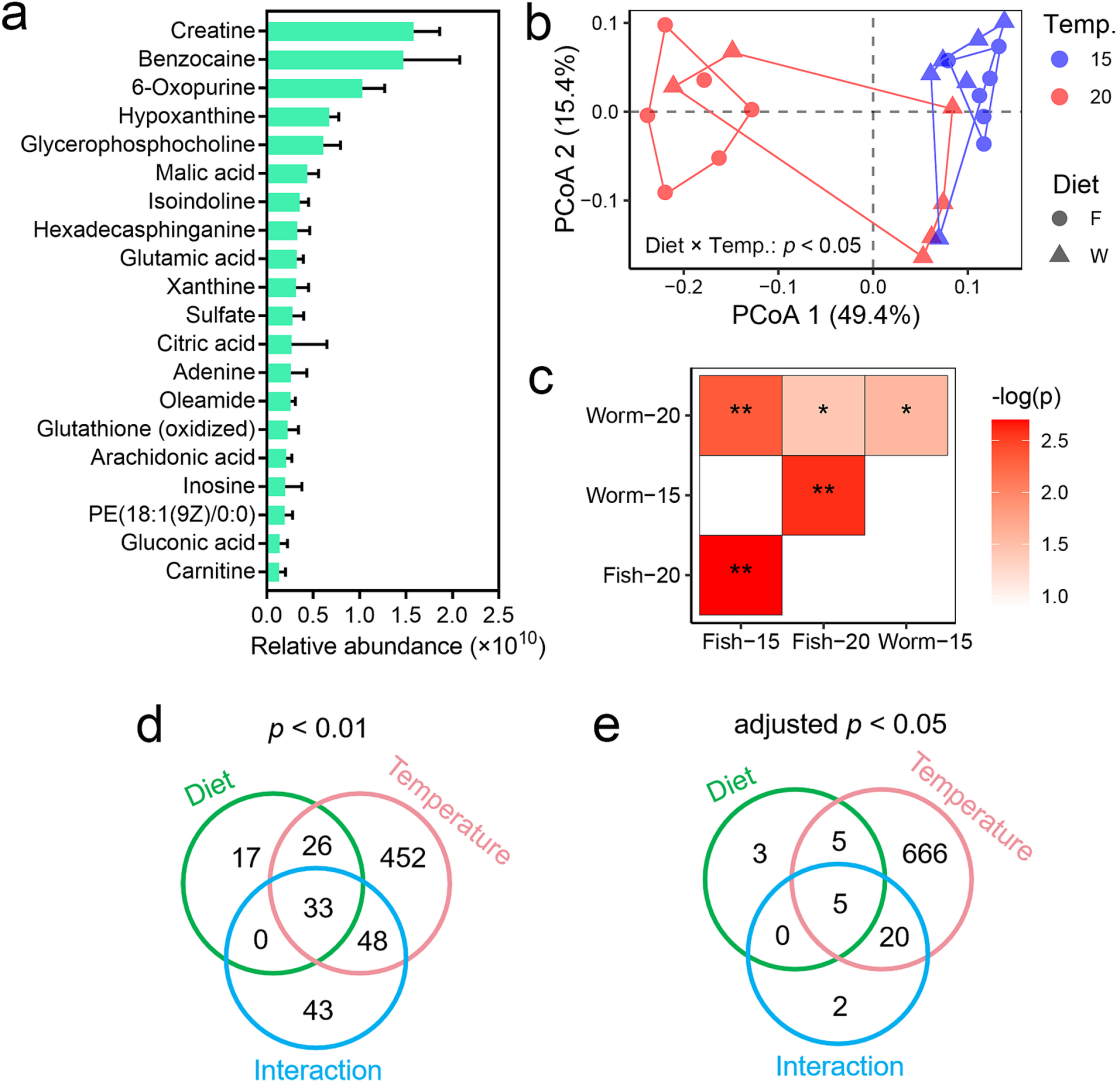
Influences of temperature and diet on the liver metabolome of *A. davidianus*. **(a)** Primary signatures of liver metabolic profiles, represented by the 20 metabolites with the highest relative abundances (based on molecular ion peak area). Please note that the relative peak area does not indicate the actual molar concentrations of the metabolites. **(b)** PCoA scatter plot illustrating dissimilarities in metabolic profiles among groups (Bray–Curtis distances). The inter-group difference was examined with multi-factor PERMANOVA, which indicated a significant interactive effect between temperature and diet on liver metabolome. **(c)** Heatmap presenting the pairwise differences in liver metabolome between groups. ***, adjusted *p* < 0.001; **, adjusted *p* < 0.01; *, adjusted *p* < 0.05 (PERMANOVA and BH correction). **(d,e)** Venn plots showing the number of differential metabolites at different threshold of significance (two-way ANOVA and BH corrections).

The liver of *A. davidianus* larvae contained high levels of creatine, malic acid, glutamic acid, and citric acid (Figure 2a), all of which are closely associated with energy metabolism. Notably, we found that temperature and diet had significant interactive effects on liver metabolome as a whole (*p* < 0.05, PERMANOVA; Figure 2b). Pairwise PERMANOVA analyses further revealed significant differences in liver metabolomes across most pairwise combinations of temperature and diet groups (Figure 2c). Two-way ANOVA was performed to identify differential metabolites (*p* < 0.01 or adjusted *p* < 0.05) associated with temperature, diet, and their interactive effects (the numbers of differential metabolites is detailed in Figures 2d,e). The number of temperature-related differential metabolites is much higher than that of diet-related metabolites, indicating that temperature had a greater impact on metabolic profiles than diet.

Given the observed significant interactive effects between temperature and diet, we conducted separate analyses to evaluate the impact of diet on the liver metabolome at each temperature, as well as the impact of temperature on the liver metabolome within each dietary condition. When comparing diets, larvae at 20°C exhibited more pronounced metabolic differences between dietary groups compared to those at 15°C (Supplementary Figures S1a,b). Several differential metabolites were identified between the diet groups at both temperatures (*p* < 0.05, Student’s *t*-test), primarily lipids such as omega-3 arachidonic acid, phosphatidylserine (PS), and phosphatidylcholine (PC) (Supplementary Figure S1c). These results underscore the significant impact of diet on lipid metabolism in *A. davidianus* larvae.

**Figure 3:**
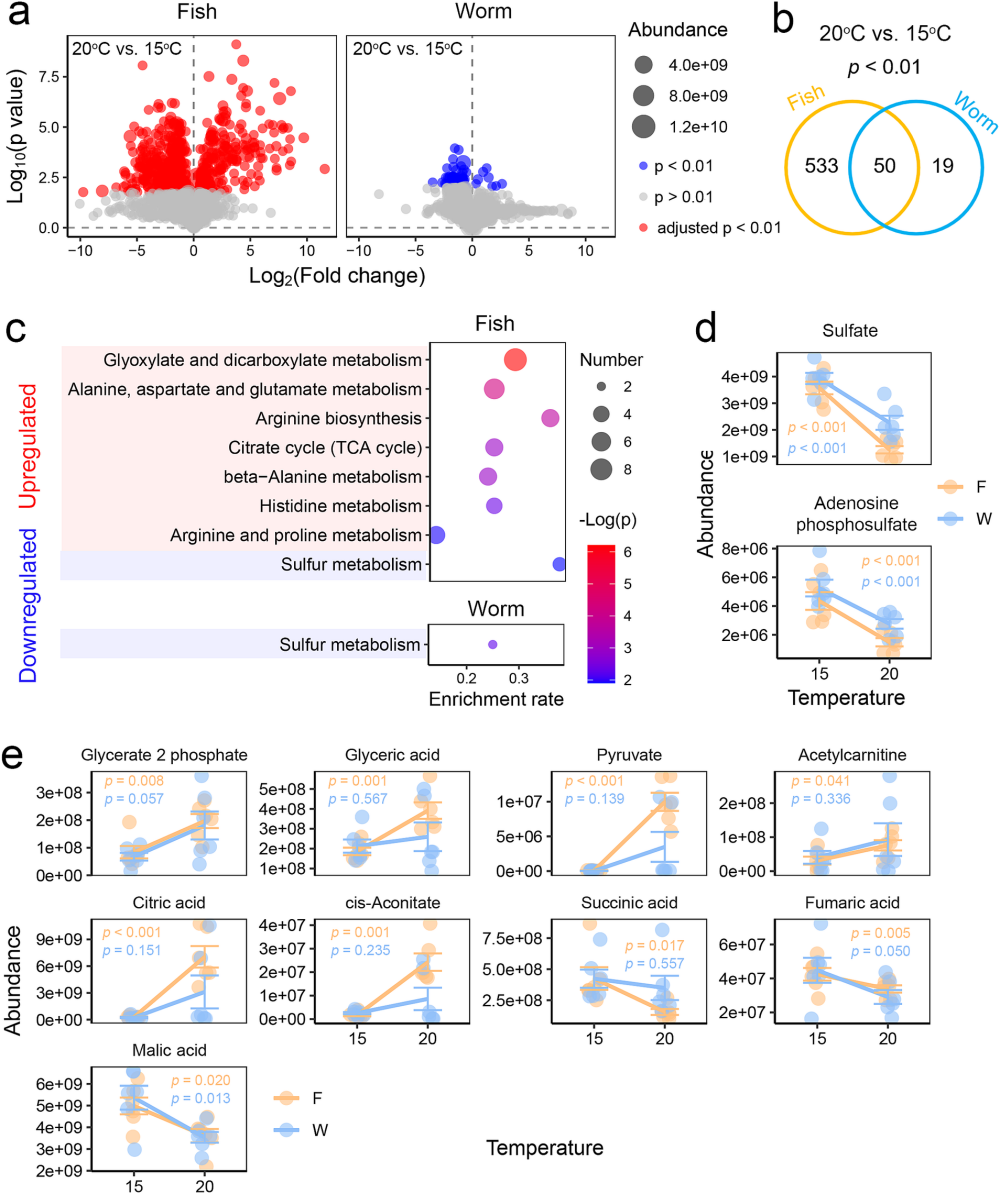
Influence of diet on thermal response in the liver. **(a)** Volcano plots illustrating temperature-induced variations in the liver metabolome of fish-fed (left) and worm-fed (right) individuals. Data were analyzed using Student’s *t*-tests with BH corrections (20°C vs. 15°C). **(b)** Venn diagram displaying the number of specific and common differential metabolites between the fish and worm groups. **(c)** Results of enrichment analysis based on temperature-related differential metabolites (*p* < 0.01, Student’s *t*-tests) for each diet group (20°C vs. 15°C). Only KEGG items with adjusted *p* < 0.05 in enrichment analysis are shown. **(d)** Dot plots depicting the variations of metabolites involved in sulfur metabolism, a metabolic pathway that varied with temperature in both fish- and worm-fed individuals. **(e)** Dot plots illustrating the variations of metabolites involved in the TCA cycle and its upstream pathways, which demonstrated more significant thermal responsiveness in fish-fed individuals. Inter-group variations in metabolic abundance were examined using two-way ANOVA. The results of the statistical analyses (Student’s *t*-tests) for different diets are represented using distinct colors.

When comparing temperatures, fish-fed individuals exhibited greater metabolic variation with temperature than worm-fed individuals (Figures 3a,b). In fish-fed individuals, metabolites increased in response to elevated temperature (*p* < 0.01, Student’s *t*-test) were associated with glyoxylate and dicarboxylate metabolism, the TCA cycle, and various amino acid pathways, while those that decreased were primarily linked to sulfur metabolism (Figure 3c). In contrast, worm-fed individuals showed no significant pathway enrichment for upregulated metabolites under heat stress, while downregulated metabolites also highlighted sulfur metabolism (Figure 3c). Key sulfur metabolism components, including sulfate and adenosine phosphosulfate, decreased significantly under heat stress in both dietary groups (Figure 3d), indicating that the effects of temperature on sulfur metabolism are diet-independent. However, variations in the TCA cycle and its upstream substrate metabolism were diet-dependent, with more pronounced changes observed in fish-fed individuals (Figure 3e). Specifically, fish-fed larvae exhibited elevated levels of metabolites in the early stages of the TCA cycle and upstream of it (e.g., citric acid, cis-aconitate, glycerate-2-phosphate, glyceric acid, pyruvate, acetylcarnitine) at higher temperatures (Figure 3e). In contrast, metabolites in the later stages of the TCA cycle (e.g., succinic acid, fumaric acid, malic acid) were reduced in these larvae. These findings indicate that energy metabolism in the liver of *A. davidianus* larvae is a target of both temperature and diet. Given the TCA cycle plays a role in heat susceptibility in *A. davidianus* larvae (Zhu et al., 2022b), this pathway may potentially mediate the impact of diet on their thermal performance.

### 3.2 Effects of temperature and diet on the gut microbial composition

**Figure 4:**
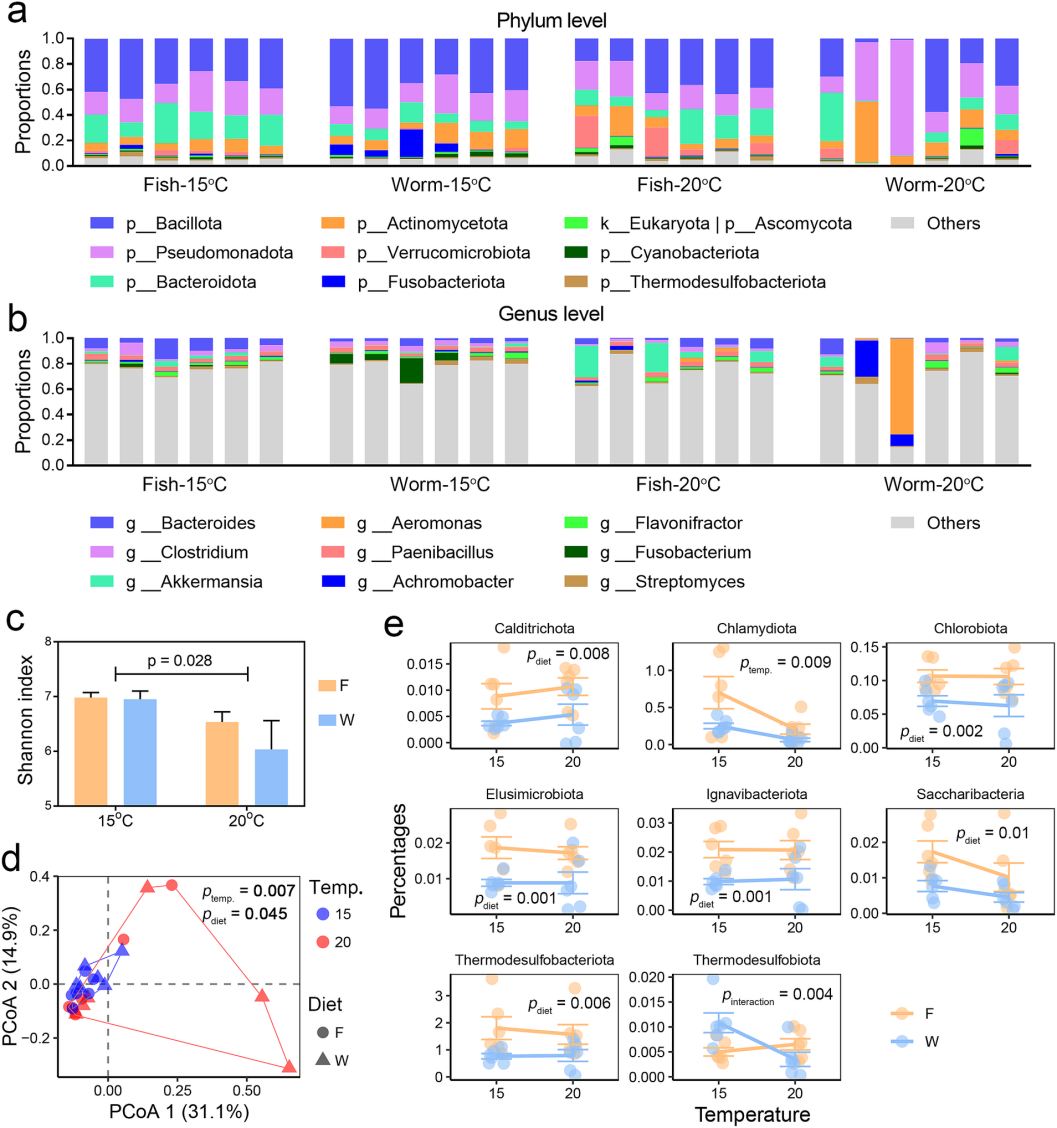
Comparative analysis of gut microbial composition. **(a,b)** Microbial composition at the phylum **(a)** and genus **(b)** levels, displaying only the 10 most abundant taxa. **(c)** Variation in gut microbial alpha-diversity across groups, with inter-group differences assessed by two-way ANOVA. **(d)** PCoA scatter plot illustrating dissimilarities in microbial composition at the species level (based on Bray-Curtis distances) between groups and samples. Multi-factor PERMANOVA (Type II sums of squares) was used to evaluate the effects of temperature and diet on gut microbial beta-diversity. **(e)** Dot plots showing the relative abundance of differentially abundant microbial phyla between groups, with significance determined for temperature, diet, or their interaction (*p* < 0.01, two-way ANOVA).

The predominant microbial taxa identified in *A. davidianus* larvae were Bacillota, Pseudomonadota, Bacteroidota, and Actinomycetota at the phylum level (Figure 4a), and *Bacteroides* (pivotal genus in the gut microbiome), *Clostridium* (significant to gut metabolism but can be pathogenic), *Akkermansia* (commonly beneficial bacteria), and *Aeromonas* (including numerous opportunistic pathogens) at the genus level (Figure 4b). Heat stress resulted in a reduction in microbial alpha diversity, while no significant differences in alpha diversity were detected between dietary groups (Figure 4c).

Both temperature and diet influenced the gut microbiota composition, with temperature exerting a more substantial effect. The microbiome profile, as a whole, was not affected by the interactive effects of temperature and diet (Figure 4d and Supplementary Figure S2). Two-way ANOVA were performed to screen the differential microbes (*p* < 0.05) between treatment groups. In response to elevated temperature, the relative abundance of the phylum Chlamydiota decreased, alongside reductions in genera such as *Aquifex*, *Fastidiosipila*, *Beijerinckia* (nitrogen-fixing bacteria), *Methylocystis* (methane oxidation), *Alcanivorax* (hydrocarbon-degrading bacteria), and *Turbidovirus* (bacteriophages). In contrast, the phage genus *Kuttervirus* (bacteriophages) exhibited an increase in relative abundance (Figure 4e and Supplementary Figure S3). Dietary influences were also evident: larvae fed a fish-based diet showed a higher relative abundance of the phyla Calditrichota, Chlorobiota, Elusimicrobiota, Ignavibacteriota, Saccharibacteria, and Thermodesulfobacteriota. Additionally, increases were observed in the genera *Fastidiosipila* (also affected by temperature), *Micavibrio* (predatory bacteria), *Prosthecochloris* (sulfur-metabolizing bacterium), *Endomicrobium* (intracellular bacterium), and *Desulfococcus* (sulfate-reducing bacteria) compared to those fed a worm-based diet (Figure 4e and Supplementary Figure S3). Notably, the relative abundance of a few bacterial taxa was affected by the interactive effects of temperature and diet, including Thermodesulfobacteriota (sulfur-reducing bacteria), *Anoxybacter* (fermentative bacteria), *Alkaliphilus*, and *Thiosulfatimonas* (sulfur-reducing bacteria). These results suggest that the relative abundance of individual microbes is jointly influenced by temperature and diet, positioning them as potential targets for diet to modulate the temperature effects on the gut. However, the link between temperature effects on microbial composition and heat susceptibility in *A. davidianus* larvae requires further functional analyses and correlation analyses with the liver metabolome.

### 3.3 Effects of temperature and diet on the gut microbial function

**Figure 5:**
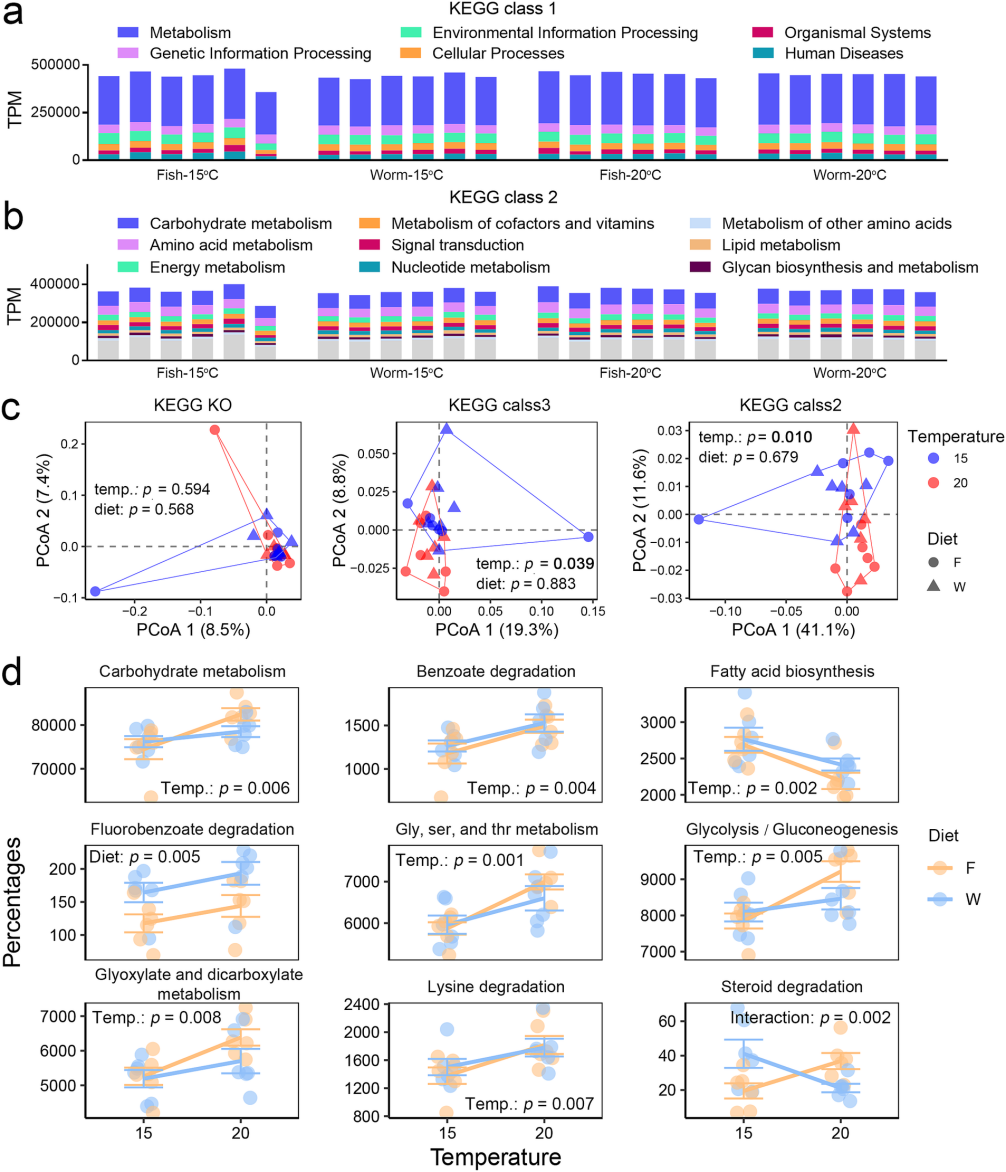
Comparative analysis of gut microbial functions. **(a,b)** Proportions of functional categories at KEGG Class 1 **(a)** and Class 2 **(b)** levels. **(c)** PCoA scatter plots illustrating dissimilarities in microbial functions among groups at KO, KEGG Class 3, and KEGG Class 2 levels, based on Bray–Curtis distances. Multi-factor PERMANOVA (Type II sums of squares) was used to assess the effects of temperature and diet on gut microbial beta-diversity. **(d)** Dot plots showing the relative abundance of differentially abundant metabolic pathways (KEGG Classes 2 and 3) between groups, with significance determined for temperature, diet, or their interaction (*p* < 0.01, two-way ANOVA).

The majority of the metagenomic functions in *A. davidianus* gut microbiota are focused on metabolic processes (Figure 5a), particularly carbohydrate metabolism, amino acid metabolism, energy metabolism, and the metabolism of cofactors and vitamins (Figure 5b).

Compared to microbial composition, microbial functions are relatively stable with respect to variations in temperature and diet. Temperature had minimal impact on gut microbial functions at the gene level (KO) but became marginally significant at the pathway level (KEGG class level 3 and 2) (0.01 < *p* < 0.05, Figure 5c). Specifically, heat stress increased the relative abundance of genes involved in carbohydrate metabolism, glycohydrolysis, benzoate degradation, glycine, serine, and threonine metabolism, glycolysis/gluconeogenesis, glyoxylate and dicarboxylate metabolism, and lysine degradation (Figure 5d and Supplementary Figure S4), while decreasing genes related to fatty acid biosynthesis. In contrast to temperature, diet showed no significant impact on the overall microbial functional profiles. Only a few metabolic pathways exhibited significant difference between the two diet groups, with the worm-fed group showing a higher relative abundance of genes involved in fluorobenzoate degradation compared to the fish-fed group (Figure 5d). These results suggest that the gut microbial functions are unlikely targets for modulating the effects of temperature through diet.

### 3.4 Correlations between alterations in gut microbiota and liver metabolome

**Figure 6:**
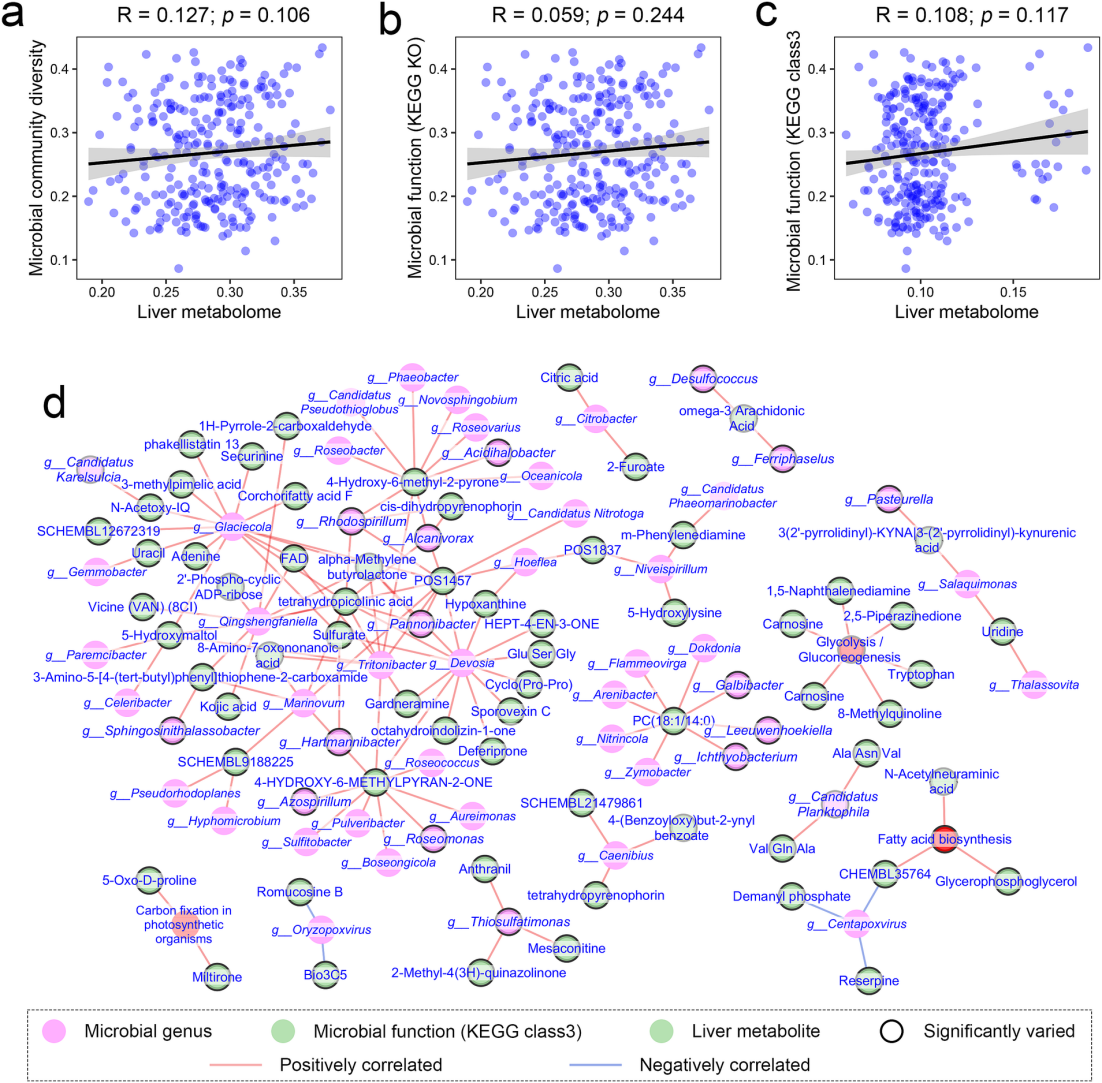
Associations between gut microbiota and liver metabolome in response to temperature variations. **(a–c)** Dot plots illustrating the correlations (Spearman correlations) between the liver metabolome and gut microbial composition and function. Data were analyzed using Mantel tests. **(d)** Correlation network displaying potential associations between liver metabolites and individual microbial genera and functions (KEGG Class 3). Only the relationships meeting adjusted *p* < 0.05 and absolute *R* > 0.75 were displayed (Spearman correlation and BH corrections). Green, pink, and red nodes represent metabolites, microbial genera, and microbial functions, respectively. Nodes with black borders indicate significant influences of diet or temperature on their relative abundance.

We investigated the relationships between variations in gut microbiota and the liver metabolome in response to changes in temperature and diet. Our analysis revealed no statistically significant correlation between liver metabolome similarity and gut microbiota compositional or functional similarity across samples (Mantel test: *R* = 0.127, *p* = 0.106; *R* = 0.059, *p* = 0.244; *R* = 0.108, *p* = 0.117; Figures 6a–c). This finding indicates that the compositional and functional diversity of the gut microbiota, as a whole, is unlikely to be a primary driver of variations in the liver metabolome.

However, at the level of individual metabolites or bacterial taxa, statistically significant correlations were detected (e.g., citric acid and *Citrobacter*), based on Spearman correlation and BH corrections (adjusted *p* < 0.05, |*R*| > 0.75; Figure 6d). For example, the relative level of citric acid was significantly correlated with the relative abundance of *Citrobacter*. Notably, these correlations involved metabolites or taxa varied significantly in levels with diet or temperature, suggesting potential co-variations between gut microbes and the liver metabolites under these conditions.

## 4 Discussion

Temperature and diet interactively influence the physiological performance of amphibians (Alvarez and Nicieza, 2002). For instance, variations in food availability modulate the effects of water temperature on survival, growth, and development in the larvae of the striped marsh frog (*Limnodynastes peronii*) (Courtney Jones et al., 2015), and warming induces dietary shifts in tadpoles (Carreira et al., 2016). However, the physiological mechanisms underlying these interactions remain poorly understood. This study examined the combined effects of temperature and diet on the gut microbiota and liver metabolome of *A. davidianus* larvae, aiming to elucidate the physiological pathways influencing their diet-dependent physiological responses to heat stress.

### 4.1 TCA cycle is a target mediate the influences of diet on thermal performance

Our findings highlight the TCA cycle as a key pathway underlying the differential temperature responses of *A. davidianus* larvae fed fish versus worms. In a previous study, larvae exposed to 25°C heat stress exhibited signs of resource depletion and impairments in hepatic energy metabolism, including decreased levels of glycolytic intermediates (e.g., fructose-1,6-bisphosphate, glyceraldehyde-3-phosphate), fatty acid metabolism intermediates (e.g., palmitoylcarnitine, stearoylcarnitine), and TCA cycle intermediates (e.g., citrate, cis-aconitate) (Zhu et al., 2022b). In this study, even moderate heat stress at 20°C led to a reduction in late-stage TCA cycle metabolites (malate and fumarate) in both fish-fed and worm-fed larvae, further supporting the notion that heat stress impairs energy metabolism in *A. davidianus* larvae. Given the increased metabolic demands at higher temperatures (Zhao et al., 2022), this reduction in metabolites likely reflects accelerated consumption under heat stress.

Dietary differences significantly influenced the metabolic response to heat stress (20°C in this study). In aerobic energy metabolism, the TCA cycle and its downstream oxidative phosphorylation pathways serve as central hubs for the catabolism of carbohydrates, lipids, and amino acids. Larvae fed a fish-based diet, rich in proteins and lipids, exhibited a pronounced upregulation of early-stage TCA cycle intermediates (citrate and *cis*-aconitate), glycolytic intermediates (e.g., 2-phosphoglycerate, glycerate, pyruvate), and fatty acid metabolism intermediates (acetylcarnitine). This could better maintain the energy metabolism than worm-based diet and thus explain the enhanced heat tolerance of *A. davidianus* larvae fed on fished-based diet. In addition to an energetic explanation, the TCA cycle can also regulates numerous other vital biological processes, including maintaining NADH/NADPH homeostasis, scavenging reactive oxygen species (ROS), generating ATP through substrate-level phosphorylation, mediating signaling pathways, and supplying metabolites for cellular repair (MacLean et al., 2023). Thus, differences in TCA cycle activity may influence the thermal physiology of *A. davidianus* through mechanisms beyond energy metabolism. However, further experimental validation is needed to confirm the relationship between TCA cycle activity, nutrition composition (e.g., protein, lipid, and carbohydrate proportions), and thermal performance.

In addition to the potential link between TCA cycle activity and thermal performance, another important aspect to consider is the mechanism underlying the enhanced TCA cycle response in fish-fed larvae. Two possible explanations exist. First, lipid and protein catabolism may fuel the TCA cycle more effectively than carbohydrates under thermal stress. This aligns with our previous finding that heat-stressed *A. davidianus* experienced impaired glycogen metabolism (Zhu et al., 2022b). Alternatively, the macronutrients (carbohydrates, lipids, and proteins) in the fish-based diet may be more readily catabolized than those in the worm-based diet, or other non-energy components of the fish-based diet may stimulate TCA cycle activity. It should be mention that TCA cycle metabolite levels in fish-fed larvae were comparable to those of worm-fed larvae under 15°C, indicating that fish-fed larvae do not inherently maintain higher TCA cycle activity. This indicates that the elevated TCA cycle metabolites observed in fish-fed larvae at 20°C likely reflect an adaptive response to increased metabolic demands under heat stress. One possibility is that worm-fed larvae exhibit metabolic constraints due to limited lipid and protein availability, or other metabolic components differed between diets, potentially impairing their ability to mount a similar adaptive response. Unveiling the mechanistic link between diet and TCA cycle activity under heat stress is crucial for gaining new insights into species conservation and warrants further investigation.

### 4.2 Role of gut microbiota mediating the effects of diet on thermal performance

**Figure 7:**
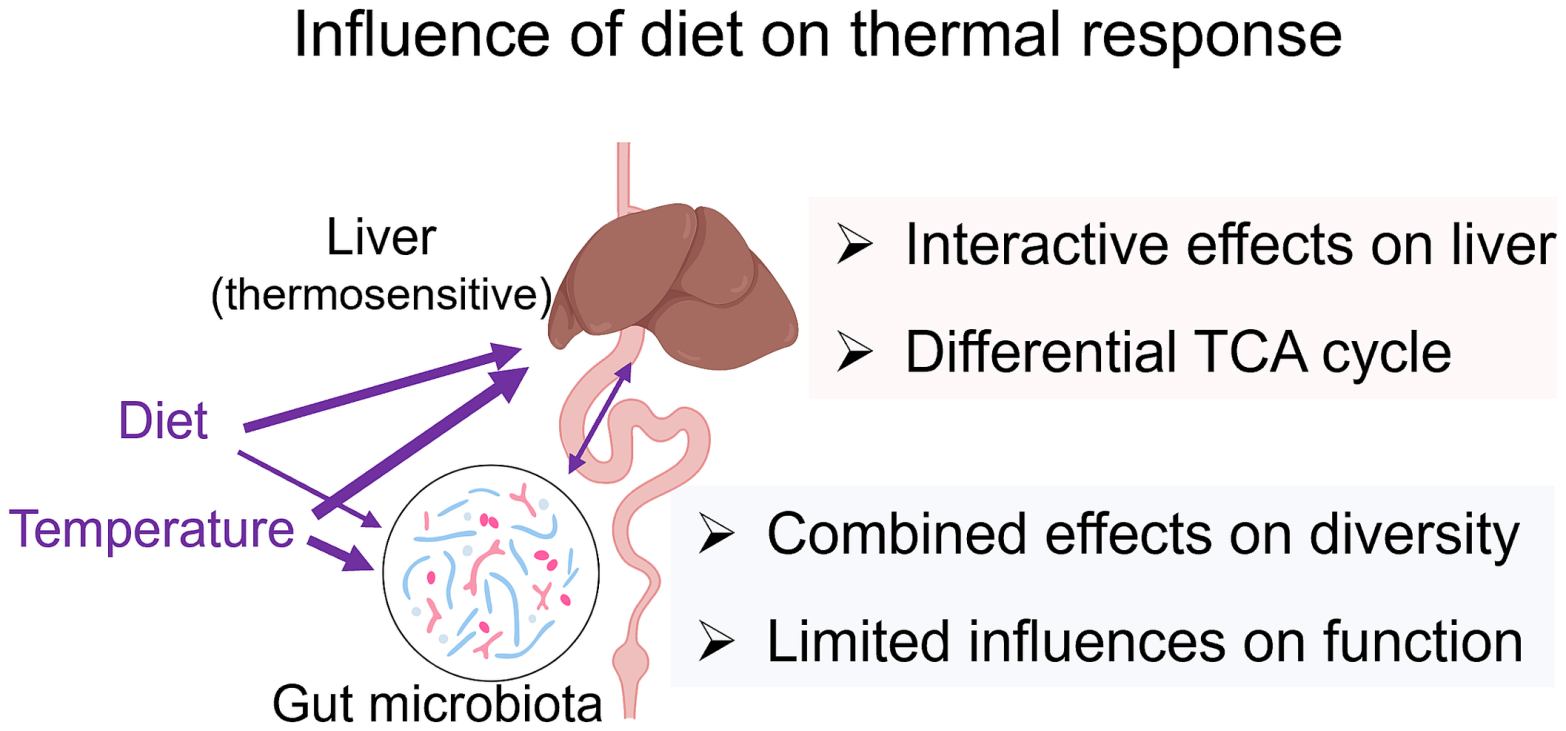
Summary of the results. The width of the arrows indicates the relative strength of influences or associations.

The gut microbiota is highly responsive to environmental variations, including changes in temperature and diet (Zmora et al., 2019; Khakisahneh et al., 2020). In *A. davidianus* larvae, both factors significantly influenced gut microbial composition, with certain microbial taxa (discussed below) being jointly regulated by temperature and diet. This suggests that the gut microbiota may serve as a potential target for dietary modulation of temperature effects on the gut. However, dietary-induced shifts in microbiota composition were not accompanied by corresponding changes in microbial metabolic function. This indicates that microbe-related metabolism is unlikely to mediate the effects of diet on thermal performance, and gut microbiota variations may not play a major role in linking diet to liver metabolism. This conclusion aligns with correlation analyses, which show no significant association between liver metabolome variations and overall gut microbial compositional or functional profiles. Together with the findings from liver metabolomics, these results suggest that diet primarily modulates liver metabolism by directly influencing host metabolism rather than through gut microbial-mediated effects (Figure 7).

Nevertheless, other potential pathways through which the gut microbiota may contribute to the interactive effects of temperature and diet on thermal performance cannot be excluded. For instance, shifts in gut microbial composition may influence gut homeostasis (e.g., immune function and metabolism) independent of functional changes, thereby indirectly affecting host thermal performance. Several bacterial groups were influenced by the interactive effects of temperature and diet, including Thermodesulfobiota at the phylum level and *Anoxybacter*, *Alkaliphilus*, *Alcanivorax*, and *Thiosulfatimonas* at the genus level. Additionally, *Fastidiosipila* was affected by the additive effects of temperature and diet. These bacterial groups are closely associated with host physiology and health (Wang et al., 2019). For instance, *Alkaliphilus* has been linked to aging in chickens (Fagundes et al., 2017) and gut homeostasis in rats (Tulyeu et al., 2019), while *Fastidiosipila* has been correlated with metabolic disorders such as type 2 diabetes in humans (Li et al., 2021). Moreover, correlation analyses reveal that some of these bacteria (e.g., *Thiosulfatimonas* and *Alcanivorax*) show significant associations between their relative abundance and liver metabolites that fluctuate with temperature or diet. These findings suggest that individual gut microbes, rather than the overall gut microbiota and metagenome, may play a role in liver metabolism of *A. davidianus* larvae. To further investigate the involvement of these microbes in thermal performance, future studies should explore the role of the gut-liver axis in heat susceptibility in *A. davidianus* larvae.

### 4.3 Conservation implications

Understanding the interplay between environmental factors and thermal performance is critical for amphibian conservation (Gangloff and Telemeco, 2018). Our findings underscore the potential of nutrient management as a conservation strategy for *A. davidianus* and other amphibian species threatened by warming. For instance, providing metabolic compounds or nutrients that are rapidly depleted under heat stress, through methods such as feed replacement or supplement addition, could be beneficial. However, such approaches are limited to captive populations or animals within carefully managed protected areas. For population conservation in natural environments, improving the accessibility and nutritional quality of prey to enhance heat stress tolerance may provide a more viable solution. Thus, future research should explore the prey preferences of *A. davidianus* under different temperature conditions and evaluate how these dietary choices influence hepatic energy metabolism (particularly the TCA cycle) and thermal tolerance. Such studies could inform efforts to optimize prey composition, thereby supporting the conservation of wild *A. davidianus* populations in specific regions.

Additionally, our study revealed correlations between the relative abundance of specific metabolites and particular microbial genera or metabolic functions. For instance, the relative abundance of citric acid in the liver of *A. davidianus* was closely associated with the relative abundance of gut microbial genus *Citrobacter* (Figure 6d). This finding suggests that modulating the abundance of certain microbial taxa could influence the metabolism of *A. davidianus*. Specifically, given that glycogen metabolism is impaired in the liver of heat-stressed *A. davidianus*, potentially contributing to energy deficiency, it may be possible to alleviate this issue by introducing bacteria with high efficiency in the conversion of carbohydrate to short-chain fatty acid transition in their gut. These insights open promising avenues for utilizing gut microbiota to improve amphibian resilience to environmental stressors (Jiménez and Sommer, 2017; Zhu et al., 2021a).

This study has several limitations. First, data at 25°C are unavailable due to high larval mortality at this temperature. The lack of data at this more stressful temperature may constrain our interpretation of key aspects, such as temperature-gradient effects on metabolism and gut microbiota, as well as dietary modulation of responses under varying heat stress conditions, potentially weakening the persuasiveness of our findings. Second, our study does not reveal the molecular mechanisms by which diet regulates liver metabolism and the TCA cycle. Addressing this gap may require isotope-labeled omics approaches for deeper insights. Third, while metagenomic data provide valuable information on microbiota composition and potential functions, they do not fully capture actual microbial activity. Future studies integrating microbial transcriptomics and metabolomics are needed to further clarify the role of gut microbiota in mediating the interactive effects of diet and temperature on thermal performance.

## 5 Conclusion

Our results indicate that diet shift significantly influences the hepatic metabolism of *A. davidianus*, particularly by modulating the TCA cycle’s response to heat stress. Compared to the worm-based diet, the fish-based diet resulted in an enhanced TCA cycle activity, characterized by increased levels of key intermediates such as citrate and succinate, indicating enhanced oxidative metabolism and ATP production. This dietary influence represents a key molecular mechanism underlying the effects of diet on the thermal physiology of *A. davidianus*. In contrast, the gut microbiota appeared to play a less prominent role in mediating dietary effects on liver metabolism, or even on the thermal performance. However, the observed associations between hepatic metabolites and specific gut microbial taxa suggest that the gut microbiota could serve as a potential target for modulating the physiology of *A. davidianus*. Future studies should test this hypothesis and investigate whether direct modulation of the TCA cycle and gut microbiota can enhance the capacity of *A. davidianus* to cope with heat stress. Our study offers new perspectives on amphibian conservation in the face of global climate change.

## Data Availability

The datasets presented in this study can be found in online repositories. The names of the repository/repositories and accession number(s) can be found: https://ngdc.cncb.ac.cn/bioproject/browse/PRJCA033480, CRA021169.

## Footnotes

^1^ https://www.metaboanalyst.ca/home.xhtml
