# Supplementary material for: Diets shape thermal responses in Chinese giant salamanders by altering liver metabolism
Source: Front Microbiol. 2025 Mar 18;16:1546912. doi: 10.3389/fmicb.2025.1546912 (PMC11959279; doi:10.3389/fmicb.2025.1546912)
Supplement: Supplementary file 1 [file Data_Sheet_1.docx]

**Supplementary data**


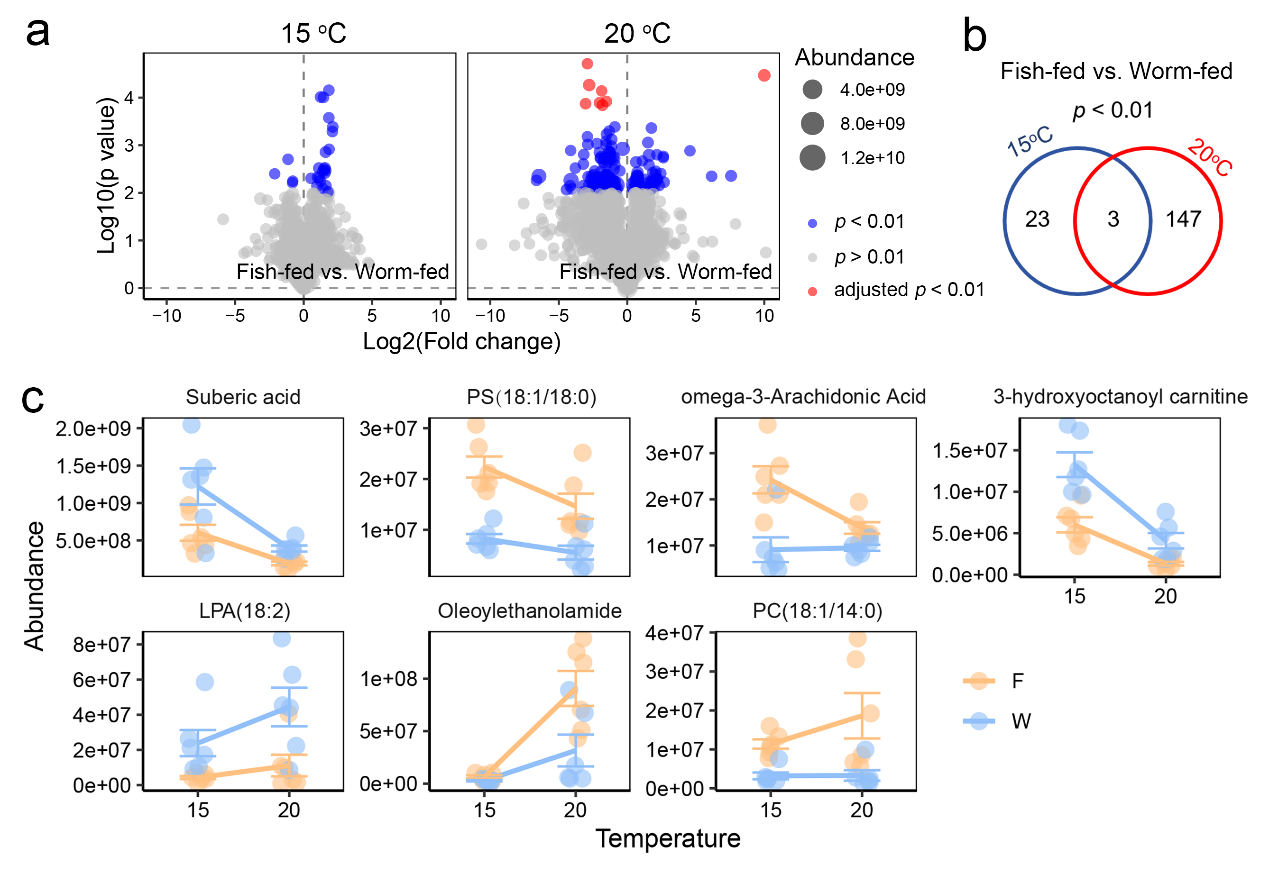


**Figure S1** **Influence of diet on liver metabolome.** (a) Volcano plots illustrating diet-induced variations in the liver metabolome at different temperatures. Data were analyzed using Student’s t-tests with BH corrections (fish-fed vs. worm-fed). (b) Venn diagram displaying the number of specific and common differential metabolites between thermal groups. (c) Dot plots illustrating the quantitative variations of lipids that significantly varied with diet at both temperatures (at threshold of *p* < 0.05, Student’s t-tests).


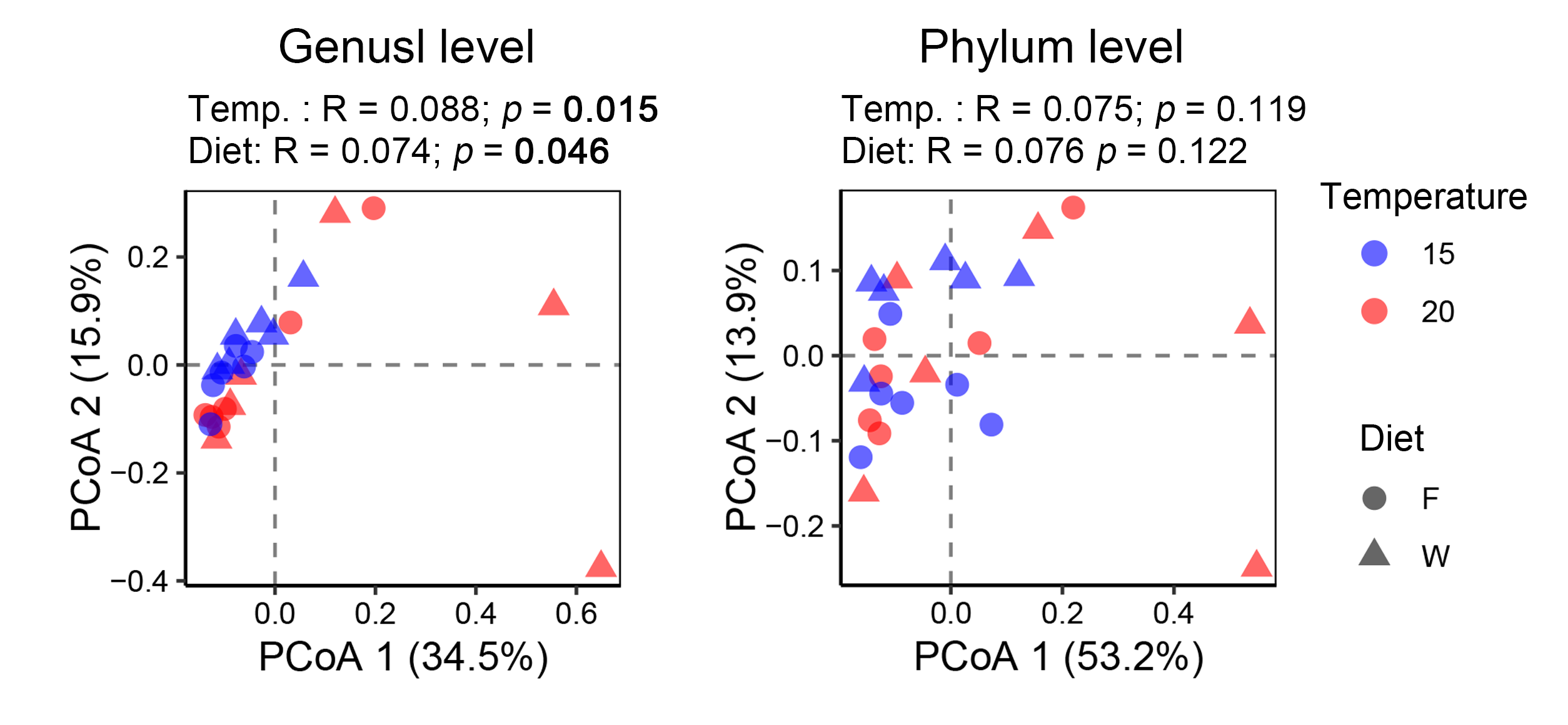


**Figure S2** PCoA scatter plot showing the dissimilarity in gut microbial composition at genus and phylum levels. The influences of temperature and diet on the microbial composition were examined with multi-factor PERMANOVA.


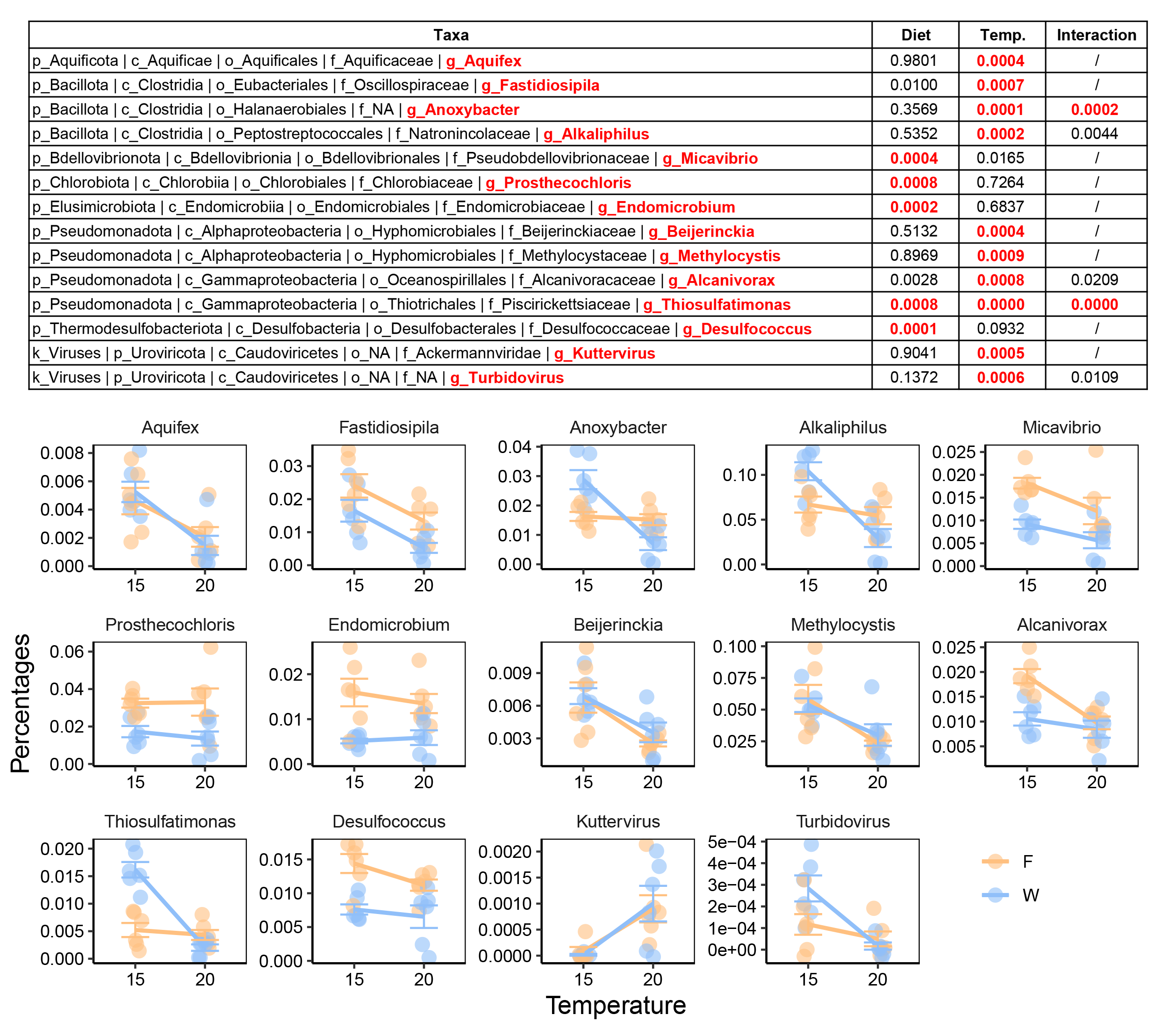


**Figure S3** Gut microbial genus whose relative abundance varied with diet or temperature significantly. The data were analyzed with two-way ANOVA. We used red color to highlight the *p* values < 0.01.


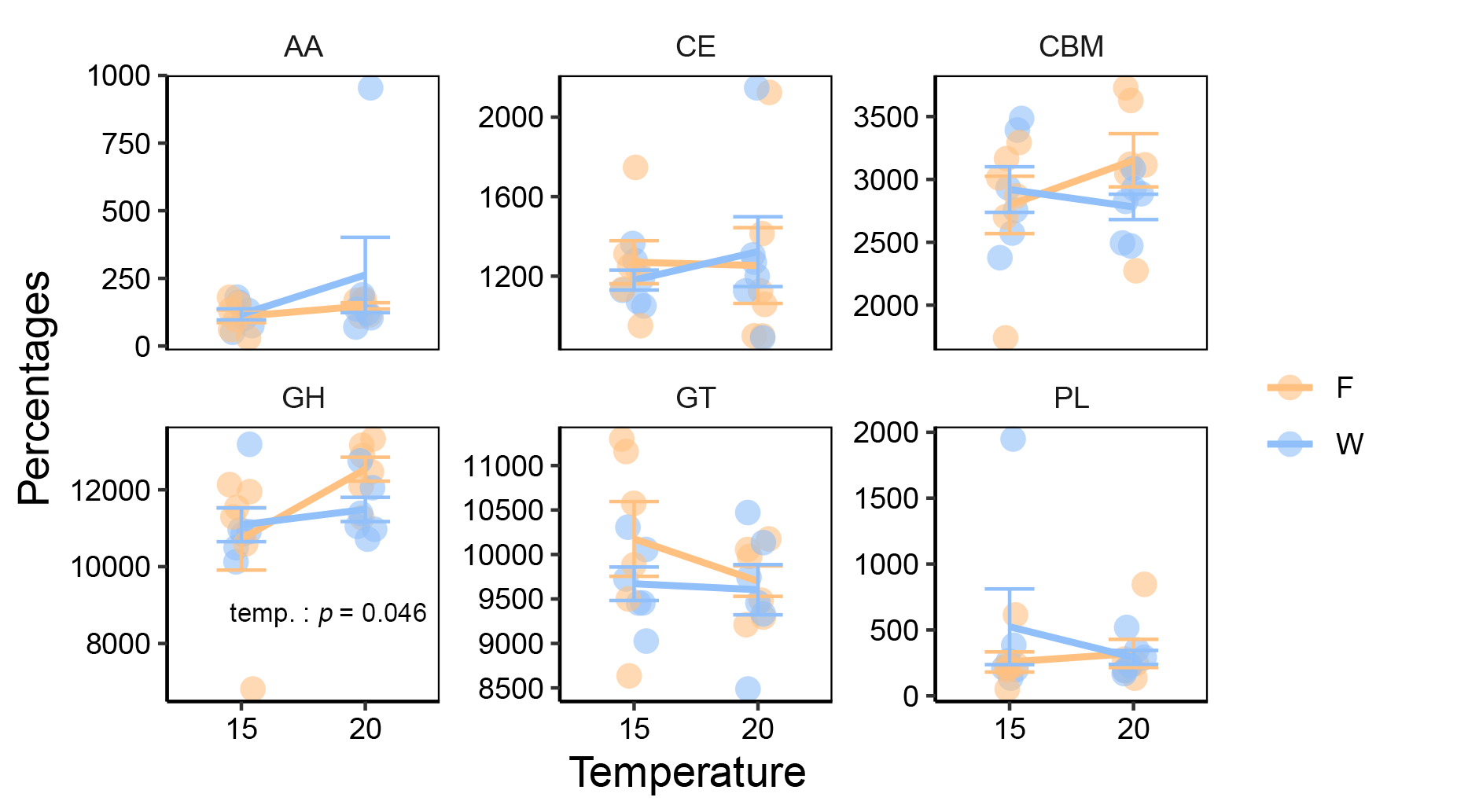


**Figure S4** Dot plots presenting the variations in the relative abundance of carbohydrate-active enzymes (module level). The data were analyzed with two-way ANOVA, with significant effects noted in the plots. AA, auxiliary activities; CE, carbohydrate esterases; CBM, carbohydrate-binding modules; GH, glycoside hydrolases; GT, glycosyl transferases; PL, polysaccharide lyases.
